# Effectiveness of the National Program of Complementary Feeding for older adults in Chile on vitamin B12 status in older adults; secondary outcome analysis from the CENEX Study (ISRCTN48153354)

**DOI:** 10.1186/1475-2891-12-124

**Published:** 2013-09-09

**Authors:** Hugo Sanchez, Cecilia Albala, Lydia Lera, Alan D Dangour, Ricardo Uauy

**Affiliations:** 1 Nutrition and Public Health, Aging, and Genetic Epidemiology Unit, Institute of Nutrition and Food Technology, University of Chile, Avda. El Libano 5524, Casilla 138–11, Santiago, Chile; 2 Department of Population Health, Faculty of Epidemiology and Population Health, London School of Hygiene & Tropical Medicine, London, UK

**Keywords:** Older people, Fortified foods, Nutrition programme, Vitamin B12

## Abstract

**Background:**

Older people are at increased risk of vitamin B12 deficiency and the provision of fortified foods may be an effective way to ensure good vitamin B12 status in later life.

**Aim:**

To evaluate the effectiveness of a vitamin B12 fortified food provided by a national program of complementary food for older people on plasma vitamin B12 levels.

**Subjects and methods:**

A random sub-sample of 351 subjects aged 65-67y from a large cluster randomised controlled trial provided blood samples at baseline and after 24 months of intervention. The intervention arm (10 clusters 186 participants) received a vitamin B12 fortified food designed to deliver 1.4 μg/day, while the control arm did not receive complementary food (10 clusters, 165 participants). Serum vitamin B12 and folate levels determined by radioimmunoassay were used to estimate the effect of intervention on vitamin B12 levels, adjusting for baseline levels and sex.

**Results:**

Attrition at 24 months was 16.7% and 23.6% in the intervention and control arms respectively (p = 0.07). Over 24 months of intervention, mean (95% CI) serum vitamin B12 decreased from 392 (359–425) pmol/dL to 357 (300–414) pmol/dL (p < 0.07) in the intervention arm and from 395 (350–440) pmol/dL to 351 (308–395) pmol/dL in the control arm. There was no significant effect of the intervention on folate status.

**Discussion:**

Our findings suggest that foods fortified with 1.4 μg/daily vitamin B12 as provided by Chile’s national programme for older people are insufficient to ensure adequate vitamin B12 levels in this population. Chile has a long and successful experience with nutrition intervention programs; however, the country’s changing demographic and nutritional profiles require a constant adjustment of the programs.

## Introduction

The ageing process of the Chilean population involve multiple repercussions on public health [1,2]. The gradual deterioration of physical and mental health conditions that accompanies ageing is a result of genetic and environmental interactions, including lifestyle, dietary habits, physical activity, and the presence of disease. Nutrition plays an important role in modulating changes induced by aging on various organ and body functions. Older people are vulnerable to multiple micronutrient deficiencies, especially vitamin B12 [3].

Vitamin B12 (B12) and folic acid are essential dietary constituents that regulate key metabolic pathways required for myelin and neurotransmitter synthesis; they also contribute to red blood cell replication and maturation [4].

Liver B12 stores are sufficient to meet adult needs for several months since daily requirements are low (2.4 μg/day) and manifestations of deficiency are apparent only after an extended dietary inadequacy (1 to 2 years) [5].

In Chile, the prevalence of B12 deficit in older adults is high with estimates ranging from 25.4% to 54.1% [6-8]. The prevalence is greater in men than women [6] and increases with advancing age [5].

In 1998 the Chilean Ministry of Health established a program for older adults designed to maintain health and activity levels in later life, reduce acute morbidity and functional decline, and decrease health inequalities [9,10]. The program includes health promotion, illness prevention, and poverty reduction initiatives [11], and a complementary feeding program for older people (called PACAM) [12], which aims to promote adequate nutrition across the life course. Since 1999, PACAM has delivered foods to adults over the age of 69 who are beneficiaries of the National System of Health Services [13] providing 1.7ug/day of vitamin B12. Since 2000, wheat flour has been mandatorily fortified in Chile with 2.0-2.4 mg Folic Acid/Kg. Bread is an important food staple in Chile and this programme is estimated to deliver 200–400 μg of folate per day to older people [14,15].

The primary objective of this study was to evaluate the effectiveness of the national complementary feeding program for older people (PACAM) on serum B12 levels in older adults. Secondary outcomes of interest include serum folate levels and relevant haematological parameters.

### Subjects and methods

This is a sub-study of the CENEX study [16] which was a cluster randomised controlled trial design to evaluate the cost effectiveness of a 2-y nutritional supplementation (PACAM) and/or physical activity program delivered at the community level to 2799 eligible subjects aged 65.0–67.9 y in a factorial design with four distinct study arms: Nutrition intervention (PACAM) alone, Exercise alone, Nutrition plus exercise and Control [17]. Exclusion criteria of the CENEX study included unable to walk unaided, unplanned 3-kg weight loss over 3 months, planning to move house within 12 months, already enrolled in the national PACAM program or reporting a current consumption of PACAM program supplement and cognitive impairment defined as a score < 13 using a 19-item Mini Mental State Examination (MMSE) [18] and scoring < 6 in the PfefferActivities) [19,20].

At baseline, socioeconomic characteristics, history of chronic diseases and self-reported health status (SRH) were registered. Anthropometric measurements and physical performance were assessed as previously described [17]. A sub-sample of 125 randomly selected participants per group was invited for the assessment of blood indicators under fasting conditions. Blood samples were obtained from 491 individuals at baseline and from 394 participants after 2-y of intervention. The CENEX study was approved by ethics committees at Institute de Nutrition and Technology of Foods (INTA; University of Chile), Ministry of Health (Government of Chile), and London School of Hygiene & Tropical Medicine (LSHTM; University of London). All study participants provided full informed written consent before being enrolled in the study.

The intervention comprised 1 kg/month of a dried soup powdered (made of a mixture of grains and vegetables) and 1 kg/month of a powdered milk drink (made of low-lactose milk), both of which were fortified with vitamins and minerals (Table 1). The recommended daily dose is 25 g/day of dried soup and 50 g/day powered milk, both products providing jointly 1.7 ug/day of vitamin B12; approximately 58% of the daily reference nutrient intake (2.4 μg/day) [21].

**Table 1:** Nutritional composition of fortified foods

	**Cream soup**		**Milk drink**	
**Nutrient content per serving**	**100 g**	**1 SERVING (50 g)**	**100 g**	**1 SERVING (25 g)**
		% RDA*		% RDA*
Energy Kcal	400.0 kcal	-	400.0	-
Protein g	13.0	-	18.0	-
lipids g	11.0	-	10.0	-
linoleic acid g	-		1.8	-
Carbo-hydrates g	62.3	-	59.5	-
Total dietary fiber g	6.2	-	1.0	-
**VITAMINS**				
Vitamin A (ug RE)^1^	240.0	15	800.0	25
Vitamin D (ug)^2^	1.5	15	16.0	80
Vitamin E (mg TE)^3^	4.0	20	32.0	40
Vitamin C (mg)	30.0	25	180.0	75
Vitamin B1 (mg)	0.35	12.5	0.8	14
Vitamin B2 (mg)	0.40	12.5	1.6	25
Niacin (mg) NE^4^	4.5	12.5	10.0	14
Vitamin B6 (mg)	1.0	25	1.6	20
Folate (ug)	100.0	25	80.0	10
Vitamin B12 (ug)	1.4	25	2.8	70
**MINERALS**				
Calcium (mg)	400.0	25	1000.0	31
Phosphorus (mg)	400.0	25	800.0	25
Magnesium (mg)	150.0	25	300.0	25
Iron (mg)	5.0	25	5.6	10
Zinc (mg)	6.0	25	12.0	20

*% of Recommended Daily Allowance per the Food Code 1 ug RE = ug retinol equivalent; 2 ug = ug of cholecalciferol; 3 mg TE = mg tocopherol equivalent; 4 mg NE = mg niacin equivalent.

Quantities expressed per 100 g dry product and per product serving [12].

Fasting venous blood sample were collected by study nurses. All blood samples were stored at −80°C and processed at the end of the study. The serum B12 and folate levels were determined in duplicate using radioimmunoassay (Dual Count Solid phase no boil Assay Siemens, LA). The red blood cell analysis included haemoglobin (Hb; g/dL), haematocrit (Hct;%), and median corpuscle volume (MCV; fL); these parameters were determined using an electronic particle counter (Cell-Dyn Model 1700 (Abbott Diagnostics, Abbott Park, IL).

#### Statistical analysis

We evaluated the groups’ compliance with PACAM intake by counting the number of PACAM products retrieved monthly from the health centre, with 12 retrievals considered 100% compliance (i.e., the collection of 2 kg in 12 of 24 mo), as systematically documented in the records kept by health centre auxiliary nurses.

The reference values proposed by Allen et al. [22] were used to categorise B12 status as deficient <148 pmol/dL, as subclinical deficient with levels between 148 and 221 pmol/dL, and as normal with levels >221 pmol/dL. We excluded participants from analysis of B12 status if they presented with baseline B12 values >680 pmol/L, as such levels suggest that the participants used vitamin supplements [23]. For folate, values below 7 nmol/L were considered deficient, 7 to 46 nmol/L as normal, and values >46 nmol/L as supraphysiological levels [23,24]. The diagnosis of anaemia was made for haemoglobin values <13 g/dL in men and <12 g/dL in women [7]. Microcytosis and macrocytosis were considered for values <80 fL and >100 fL, respectively [25].

The five self-reported health status (SRH) response categories were regrouped into good (excellent, very good and good) fair and poor.

At baseline and post-intervention, mean (95% CI) or median (IQR: p25-p75) plasma levels and status (percentage, 95% CI) of B12 and folates are reported.

The differences between baseline and post-intervention mean plasma levels of B12 and folate are reported as mean differences and 95% CI adjusted for baseline levels and accounting for the clustered design in an intention to treat analysis. The statistical significance of the differences was analysed by Wald Test. All statistical analyses were carried out in STATA 12.0.

## Results

**Figure 1:**
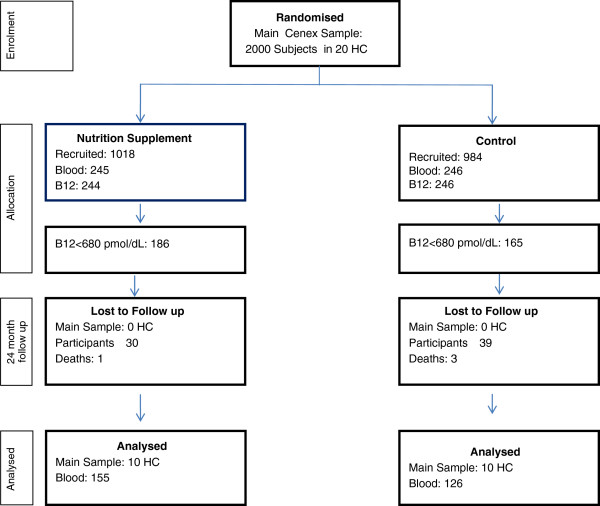
Flow-chart for study.

From the 491 subjects initially evaluated, 490 blood samples allowed the measurement of vitamin B12 levels, from which 351 met the inclusion criterion for plasma B12 levels <680 pmol/dL: 186 (28% male) in the intervention arm and 165 (30.9% male) in the control arm. At 24 months, 31 participants from the PACAM group (16.7%) were lost to attrition, and 39 were lost from the control group (23.6%) (p = 0.07). A flow diagram is shown in Figure 1.

Participants collected 68% of total available food and adherence was 75%. Only one person did not comply at all and 61,6% complied most of the time.

The baseline characteristics split by arm of intervention and gender are presented in Table 2. The intervention and control arms were well matched for age, gender, educational attainment, self-perception of health and haematological parameters at baseline.

**Table 2:** Baseline characteristics of study groups

	**Pacam**			**Control**		
	**Men**	**Women**	**Total**	**Men**	**Women**	**Total**
**n**	**52**	**134**	**186**	**51**	**114**	**165**
**Age**						
*Median* (*IQR*)	66(65-67)	66(65-67)	66(65-67)	66(65-67)	66(65-67)	66(65-67)
**Education**						
0-5 years%	18.0	34.9	30.1	19.6	33.3	28.9
6-10 years%	38.0	46.8	44.3	51.0	47.2	48.4
>10 years%	44.0	18.2	25.6	29.4	19.4	22.6
**Self-reported health status**						
*Good to excellent*; *n* (%)	25(50.0)	57(42.5)	83(44.6)	26(51.0)	45(39.5)	71(43.0)
*Fair*; *n* (%)	24(46.1)	69(51.5)	93(51.5)	23(45.1)	60(52.6)	83(50.3)
*Poor*; *n* (%)	2(3.9)	8(6.0)	10(5.4)	2(3.9)	9(7.9)	11(6.7)
**Vitamin B12 (pmol/L)**	337.3	408.0	386.5	355.0	414.8	385.2
*Median* (*IQR*)	(250.1-438.3)	(279.8-535.2)	(290.2-524.4)	(246.3-474.3)	(294.6-576.7)	(270.9-513.9)
**Folate (nmol/L)**	37.2	39.2	39.0	37.4	38.4	37.7
*Median* (*IQR*)	(33.7-43.7)	(32.6-43.7)	(32.9-43.7)	(29.6-41.1)	(32.3-42.6)	(31.5-41.7)
**Hct (%)**	43.7	39.0	40.1	43.7	39.6	40.6
*Median* (*IQR*)	(41.3-45.3)	(37.0-40.7)	(37.8-42.6)	(41.4-45.8)	(37.6-41.3)	(38.4-42.6)
**Hb (gr/dL)**	14.9	13.1	13.5	14.9	13.3	13.7
*Median* (*IQR*)	(14.0-15.3)	(12.6-13.9)	(12.8-14.4)	(13.8-15.7)	(12.6-13.9)	(12.9-14.5)
**MCV(fL)**	90	89	89	89	89	89
*Median* (*IQR*)	(88-93)	(86-91)	(87-91)	(86-92)	(86-91)	(86-92)

(IQR: p25-p75).

In Table 3 baseline and final levels of B12 and folates are shown, along with the mean and 95% CI of the serum levels of B12 (pmol/L) and folates (nmol/L) by gender. B12 levels were significantly lower in men than women, both in the PACAM group (p = 0.02) and the control group (p = 0.05). No differences in serum B12 levels at baseline neither at 24 months were observed between PACAM and control groups. Serum B12 levels decreased between baseline and 24 months only in men in both PACAM (−39.4, p < 0.01) and control (−57.3, p < 0.06). No difference in the magnitude of the decrease between both groups was observed.

**Table 3:** Baseline and post-intervention the vitamin B-12 and folates by group and sex

	**Vitamin B12 (pmol/L)**				**Folates (nmol/L)**			
	**PACAM**		**CONTROL**		**PACAM**		**CONTROL**	
	***Baseline***	***24 months***	***Baseline***	***24 months***	***Baseline***	***24 months***	***Baseline***	***24 months***
***MEN***	52	40	51	40	64	48	66	52
Mean	347.6^1^	307.6 ^1^	374.6^2^	317.2^2^	39.7^1^	31.4^1^	37.9^2^	30.7^2^
(95% CI)	(289.2-404.9)	(264.7-350.5)	(307.8-441.4)	(277.7-356.8)	(35.7-40.1)	(29.3-33.4)	(35.7-40.1)	(27.7-33.6)
Change	-39.4		-57.3		-8.3		-7.2	
(95% CI)	((-66.1)-(-12.7))		((-3.3)-117.9)		(-11.5-(-5.2))		(-11.6-(-2.9))	
***WOMEN***	134	115	114	86	180	137	179	137
Mean	407.6	374.3	404.2	367.4	40.3^1^	31.7^1^	39.4^2^	31.1^2^
(95% CI)	(377.3-437.8)	(308.2-440.4)	(354.5-454.0)	(314.0-420.7)	(37.3-43.4)	(29.5-33.9)	(36.3-42.5)	(29.3-32.8)
Change	-33.2		-36.9		-8.6	-8.3		
(95% CI)	((-84.5)-18.0)		((-96.6)-22.8)		(-13.2-(-4.1))	(-10.9-(-5.7))		
***TOTAL***	186	155	165	126	244	205	245	189
Mean	392.0^1^	357.1^1^	394.8	351.4	40.2^1^	31.6^1^	39.0^2^	30.9^2^
(95% CI)	(358.9-425.0)	(300.0-414.2)	(349.8-439.9)	(307.8-395.1)	(37.3-43.1)	(29.7-33.6)	(36.3-41.7)	(29.1-32.8)
Change	-34.8		-43.4		-8.5		-8.0	
(95% CI)	((-73.4)-3.7)		((-97.8)-11.0)		(-12.6-(-4.5))		(-10.8-(-5.2))	

Vitamin B12 ^1,2^Differences between basal and post-intervention level: ^1^PACAM: Adjusted Wald Test: Men: p < 0.01; Total p = 0.07; ^2^CONTROL: Adjusted Wald Test: Men: p = 0.06. Folates: Differences between basal and post-intervention level: ^1^Adjusted Wald Test p < 0.01; ^2^Adjusted Wald Test p = <0.01.

No differences in serum folate levels at baseline neither at 24 months were observed between PACAM and control groups. Serum folates levels decreased significantly (p < 0.01) between baseline and 24 months in both PACAM (−8.3 nmol/L) and control (−7.2 nmol/L) groups, with no differences in the magnitude of the fall between both groups.

Table 4 presents B12 and folate status by category and sex; this shows that the frequency of B12 deficiency was similar in the two groups at baseline and after intervention. After adjusting for sex, age, and baseline B12, the multilevel model indicated no significant change in B12 after intervention for either the PACAM or control groups (data not shown). No folate deficiency at baseline or post-intervention was observed.

**Table 4:** Vitamin B-12 and folates status before and after intervention, by group and sex

	***ME***				***WOMEN***				***TOTAL***			
	**PACAM**		**Control**		**PACAM**		**Control**		**PACAM**		**Control**	
	**% (CI 95%)**		**% (CI 95%)**		**% (CI 95%)**		**% (CI 95%)**		**% (CI 95%)**		**% (CI 95%)**	
	Baseline*	24 months**	Baseline*	24 months**	Baseline*	24 months**	Baseline*	24 months**	Baseline*	24 months**	Baseline*	24 months**
***Vitamin B12 (pmol*/*L*)**												
***n***	52	40	51	40	134	115	114	86	186	155	165	126
<*148*	13.5	7.5	7.8	7.5	9.0	7.8	7.0	9.3	10.2	7.7	7.3	8.7
	(5.1-31.1)	(1.8-26.5)	(2.9-19.6)	(2.6-19.8)	(4.8-15.9)	(2.9-19.3)	(3.2-14.5)	(4.2-19.2)	(5.9-17.2)	(3.4-16.8)	(3.6-14.0)	(4.5-16.2)
*148*-*221*	7.7	15.0	9.8	17.5	6.0	13.9	5.3	16.3	6.4	14.2	6.7	16.7
	(4.1-14.0)	(9.3-23.4)	(4.3-20.8)	(9.3-30.4)	(3.4-10.2)	(9.0-20.8)	(3.0-8.9)	(9.2-27.2)	(5.1-8.1)	(9.8-20.2)	(3.6-12.0)	(11.0-24.4)
*222*-*680*	78.8	77.5	82.3	72.5	85.1	72.2	87.7	65.1	83.3	73.5	86.1	67.5
	(64.7-88.4)	(59.5-89.0)	(69.2-90.7)	(66.9-77.5)	(78.5-89.9)	(59.6-82.0)	(80.6-92.4)	(49.4-78.1)	(76.0-88.8)	(62.5-82.3)	(79.4-90.8)	(57.0-76.4)
>680**		0 (0)		2.5		6.1		9.3		4.5	7.1	
				(0.3-16.8)		(2.9-12.1)		(4.8-17.1)		(2.3-8.6)	(3.7-13.3)	
***Folates (nmol*/*L*)**												
***n***	64	48	66	52	180	157	179	137	244	205	245	189
<*7*	0	0	0	0	0	0	0	0	0	0	0	0
*7*-*46*	79.7	91.7	89.4	90.4	77.2	92.4	77.6	95.6	77.9	92.2	80.8	94.2
	(67.8-88.7)	(80.0-97.7)	(79.4-95.6)	(79.0-96.8)	(70.4-83.1)	(87.0-96.0)	(70.8-83.5)	(90.7-98.4)	(72.1-82.9)	(87.6-95.4)	(75.3-85.5)	(89.8-97.0)
>*46*	20.3	8.3	10.6	9.6	22.8	7.6	22.3	4.4	22.1	7.8	19.2	5.8
	(11.3-32.2)	(2.3-20.0)	(4.4-20.6)	(3.2-21.0)	(16.9-29.6)	(4.0-13.0)	(16.5-29.2)	(1.6-9.3)	(17.1-27.9)	(4.5-12.4)	(14.4-24.7)	(2.9-10.1)

B12* Test χ2: Men: p = 0.69; Women: p = 0.80; Total p = 0.72. **Test χ2: Men: p = 0.61; Women: p = 0.76; Total p = 0.59. Folates* Test χ2: Men: p = 0.21; Women: p = 0.96; Total p = 0.72. **Test χ2: Men: p = 0.85; Women: p = 0.25; Total p = 0.55; **High Level excluded analysis baseline.

In terms of folate, the prevalence of values >46 nmol/L was 20.6% at baseline decreasing to 6.8% at 24 months. After adjusting for sex, age, and baseline folate, the multilevel model indicated a significant change in folate after intervention for either the PACAM or control groups (data not shown).

The prevalence of anaemia was 8.3% at baseline and 7.5% at 24 months. Macrocytosis was 0.3% at baseline, increasing to 2.1% after intervention.

## Discussion

The results demonstrate that the program did not significantly improve B12 status in comparison with the control group. B12 deficiency is relatively common among older people and is associated with metabolic, haematological, cognitive, and neuroconduction problems [26]. In a study recently published by our group, we found a B12 deficit of 12% using the cut-off of <148 mpol/L and 25.4% using the cut-off of <221 pmol/L [6]. Allen obtained similar results in an American population [27] and Loikas in a Turkish population [28].

The possible causes of B12 deficiency in the older adult population fall into two main categories: a) gastric atrophy associated with aging, which impedes the absorption of B12 from foods due to a lack of acid [29,30], along with decreased levels of intrinsic factor, which is an essential element for B12 absorption in the ileum; and b) low intake of B12 containing animal products [31]. Preliminary data from an on-going study [32] suggest that the intake of B12 from foods in older adults is on average 5.4 μg/day. The average intake of foods delivered by PACAM is 25 g/day of powdered milk drink and 3 portions/week of dried soup. Therefore the vitamin B12 intake provided by the programme is about 0.7 μg/day, thus resulting in an intake of 6.1 μg/day. Therefore, considering the recommended daily requirements (2.4 μg/day) for older people it would seem that the deficiency observed in Chile is due primarily to gastric atrophy.

One of the great challenges in nutrition program design at a population level is defining the best course of action for addressing B12 values below the acceptable limit (148 pmol/L).

Studies of a Latino population residing in the United States have demonstrated normalised plasma B12 levels after adding fortified foods or supplements (F/S) >500 μg/day to the diet. In addition, it has been found that F/S in the crystalline form of the vitamin is the most effective, as it is well absorbed by individuals with and without gastric mucosa atrophy [33].

A possible weakness of this study was that the intake of PACAM products was not specifically quantitated; however, considering that the study was an effectiveness evaluation, no assessment of PACAM intake beyond the usual controls of the program was considered. Another potential weakness was not establishing the usual intake of B12, but Chilean data suggest that the average intake of B12 in older adults is above the daily-recommended allowance.

After the intervention, the folate levels decreased in both groups, showing that PACAM fortification with folic acid has little or no effect. Taking into account that the absorption of folic acid is not influenced by age, and bread consumption in Chile is very high in all groups of age, it is possible to postulate that this decrease is due to a reduction in the amount of folic acid in wheat flour during the years when the CENEX study was carried out, as described by Castillo [15].

In Chile, the National Program of Complementary Feeding for the Elderly over the age of 70 provides 45.8% of the daily-recommended intake of B12. Our findings show that this amount does not ensure that this population maintains adequate levels of B12. Therefore, it is important to address a number of questions regarding the nutritional programs implemented in our country: First, we should evaluate the true amount of micronutrients provided by the foods included in these programs. Second, we should evaluate the effectiveness of fortifying PACAM products with crystalline B12 in quantities greater than the RDA. Finally, we should review existing B12 recommendations for this age group.

There is an international consensus on the need to target B12 in particular in nutrition interventions for older adults if foods are universally fortified with folic acid [34].

Chile has a long and successful experience in nutrition intervention programs; however, the country’s changing demographic, epidemiologic and nutritional profile requires a constant surveillance of the nutritional programs, carrying out periodic evaluations of the programs’ impact on the target population.

The high burden of illness associated with chronic nutrition-related diseases on a global and national level significantly affects national health budgets. This makes it even more important to evaluate the interventions and identify the most cost-effective course of action [35], without losing sight of the fact that nutrition and physical activity are fundamental for maintaining an active, healthy, and autonomous lifestyle and high quality of life for older adults.

## Competing interests

The authors declare that they have no competing interests.

## Authors' contributions

HS and CA conceived the study. RU, CA, AD, HS applied for funding. All authors contributed to designing the study. HS, CA, AD, RU and LL critically reviewed and revised the manuscript. All authors read and approved the final manuscript.
